# Assessment of the proliferative, apoptotic and cellular renovation indices of the human mammary epithelium during the follicular and luteal phases of the menstrual cycle

**DOI:** 10.1186/bcr994

**Published:** 2005-02-16

**Authors:** Maria Alicia H Navarrete, Carolina M Maier, Roberto Falzoni, Luiz Gerk de Azevedo Quadros, Geraldo R Lima, Edmund C Baracat, Afonso CP Nazário

**Affiliations:** 1 Department of Gyneology, Mastology Division, Federal University of São Paulo, São Paulo, Brazil; 2 Department of Neurosurgery, Stanford University School of Medicine, Stanford, California, USA; 3 APC Pathology, São Paulo, Brazil

**Keywords:** apoptosis, Ki-67, mammary gland, menstrual cycle, TdT-mediated dUTP nick end labeling

## Abstract

**Introduction:**

During the menstrual cycle, the mammary gland goes through sequential waves of proliferation and apoptosis. In mammary epithelial cells, hormonal and non-hormonal factors regulate apoptosis. To determine the cyclical effects of gonadal steroids on breast homeostasis, we evaluated the apoptotic index (AI) determined by terminal deoxynucleotidyl transferase-mediated dUTP nick end labeling (TUNEL) staining in human mammary epithelial cells during the spontaneous menstrual cycle and correlated it with cellular proliferation as determined by the expression of Ki-67 during the same period.

**Methods:**

Normal breast tissue samples were obtained from 42 randomly selected patients in the proliferative (*n *= 21) and luteal (*n *= 21) phases. Menstrual cycle phase characterization was based on the date of the last and subsequent menses, and on progesterone serum levels obtained at the time of biopsy.

**Results:**

The proliferation index (PI), defined as the number of Ki-67-positive nuclei per 1,000 epithelial cells, was significantly larger in the luteal phase (30.46) than in the follicular phase (13.45; *P *= 0.0033). The AI was defined as the number of TUNEL-positive cells per 1,000 epithelial cells. The average AI values in both phases of the menstrual cycle were not statistically significant (*P *= 0.21). However, the cell renewal index (CRI = PI/AI) was significantly higher in the luteal phase (*P *= 0.033). A significant cyclical variation of PI, AI and CRI was observed. PI and AI peaks occurred on about the 24th day of the menstrual cycle, whereas the CRI reached higher values on the 28th day.

**Conclusions:**

We conclude that proliferative activity is dependent mainly on hormonal fluctuations, whereas apoptotic activity is probably regulated by hormonal and non-hormonal factors.

## Introduction

The cyclical transformations and cellular kinetics of the mammary gland have long been the subject of intense investigation. Interest in alterations caused by steroid actions, specifically on epithelial proliferation, has risen sharply in the past decade owing to increasing numbers of breast carcinomas in the female population [1]. There is also an immediate need for more accurate predictors of breast cancer risk, particularly in light of the various chemoprevention trials under way.

Understanding the factors and mechanisms that regulate hormone-related changes in the normal human breast is crucial, because alterations in breast structure and function during the menstrual cycle could predispose this tissue to malignant changes and hence to the development of breast cancer. The present study focuses on epithelial cells, the primary target for carcinogenesis, as demonstrated by various histological studies indicating that most breast tumors arise in the epithelial cell population [2].

Although several groups have examined the proliferative activity of epithelial cells in the normal breast throughout the menstrual cycle [3-8], an underlying limitation in these studies is the concurrent use of oral contraceptives by many of the patients studied. In addition, studies with the thymidine labeling index demonstrated that proliferative activity declines with age [7,8], thus requiring standardization of the data to reduce variability. Finally, these studies do not provide a clear explanation for the hormone-related variations in programmed cell death (apoptosis) and mitosis (proliferation) observed during the menstrual cycle.

It is well known that the mammary gland undergoes morphological modifications during the menstrual cycle. Epithelial proliferation is greatest during the luteal phase, suggesting a synergistic influence of estrogen and progesterone [9,10]. Work by Ferguson and Anderson [5] and by Anderson and colleagues [6], using a linear regression model, suggests that there are cyclical variations in epithelial apoptosis levels, with maximal apoptotic expression on the 28th day of the menstrual cycle, approximately 3 days after the mitotic peak. This coincides with decreasing levels of estrogen and progesterone at the end of the menstrual cycle. Although small temporal differences were found between the two phenomena, the authors stated that it was not possible to assume a link between mitosis and apoptosis in those studies. Because the number of cells undergoing cell death was very low, apoptotic frequency was evaluated per mammary lobule (approximately 50 lobules per case). The few apoptotic cells observed seemed to be randomly distributed in the lobule, within central and peripheral ducts. The authors also included patients in whom neither apoptosis nor mitosis could be observed, requiring mathematical manipulation of the data obtained in all cases examined in order to derive mitotic and apoptotic frequencies. Indeed, in the study by Anderson and colleagues [6], it is still unclear whether the apoptotic activity described was more intense at the end of the luteal phase because it followed increased mitotic activity in the days before or because it was independently greater during that period of the menstrual cycle.

To clarify the cyclical effects of gonadal steroids on breast homeostasis, we evaluated the apoptotic index (AI) in human mammary epithelial cells (by terminal deoxynucleotidyl transferase-mediated dUTP nick end labeling (TUNEL) staining [11]) during the spontaneous (natural) menstrual cycle and correlated it with cellular proliferation as determined by Ki-67 expression [12] during the same period. We also correlated the progesterone levels with apoptosis and proliferation irrespective of the stage of the cycle.

## Materials and methods

### Patients

The series consisted of 42 patients (age 21.8 ± 6.1 years (mean ± standard deviation), range 14 to 38 years) undergoing excision of fibroadenomas, a condition thought not be associated with an increased risk of cancer, at the Department of Gyneology, Mastology Division, Federal University of São Paulo, Paulista Medical School. Institutional review board approval was obtained and patients signed an informed consent before enrollment in the study. Inclusion criteria for entry into the study were as follows: female sex, regular menstrual cycles (intervals of 28 ± 2 days) in the previous 6 months, and with a known date of last menstrual period. Exclusion criteria were: use of hormone therapy in the previous 12 months, pregnancy, females nursing in the previous 12 months, patients with any type of endocrine pathology, or patients taking any type of medication at the time that tissue for the study was obtained.

Patients underwent a complete medical exam, ultrasonography, and fine needle aspiration with subsequent microscopic analysis to confirm that the mammary lesion was benign. All patients were randomized into two groups according to the phase of their menstrual cycle, which was determined by the date of the patient's previous as well as subsequent menstrual period: group A, follicular phase (age 23.5 ± 6.6 years (mean ± SD)); group B, luteal phase (age 20.0 ± 5.2 years). Progesterone levels were examined by radioimmunoassays, with values of 3.0 ng/ml or more considered compatible with adequate luteal activity [13].

The mammary tissue was obtained with a local anesthetic without vasoconstrictors (2% lidocaine was used), at about 1:00 pm to minimize the effects of circadian rhythms on hormone release [4,14] and on cellular kinetics [15,16]. The parenchymal mammary tissue removed (at least 1 cm from the lesion) was without any gross microscopic alterations [17].

### Histopathology, TUNEL and immunohistochemistry

After fixation in 10% formaldehyde and embedding in paraffin, tissues samples were cut into 4 μm sections, deparaffinized in xylene and hydrated in a series of ethanol washes, stained with hematoxylin and eosin, and examined for any pathological abnormalities. TUNEL staining was performed with the ApopTag Plus *in situ *Apoptosis Detection Kit (Oncor, Gaithersburg, MD, USA). Tissue sections were deparaffinized and hydrated as above, and stripped of proteins by incubation with 20 μg/ml proteinase K (42°C) for 15 min. The slices were then washed in distilled water and PBS and incubated in 3% H_2_O_2 _to remove endogenous peroxidases. After equilibration, the sections were incubated at 37°C in terminal deoxynucleotidyl transferase (TdT) enzyme and digoxigenin-labeled substrate for 1 hour. Antidigoxigenin was then applied, and detection was accomplished with diaminobenzidine substrate solution. The sections were then counterstained with methyl green, cleared, and mounted. Sections treated with DNAse I enzyme were used as positive controls (Amersham, Cleveland, OH, USA), and sections from which TdT enzyme was omitted were used as negative controls. Adjacent sections were used for Ki-67 immunohistochemical staining. Sections were deparaffinized, hydrated, incubated with proteinase K, washed in distilled water and PBS, and incubated with 3% H_2_O_2 _as above. Blocking serum was applied for 20 min and sections were then sequentially incubated in primary mouse anti-human Ki-67 antibody (1:100, clone Ki-S5; Dako, Carpinteria, CA, USA) for 1 hour, biotinylated rabbit anti-mouse IgG (Dako LSAB kit) for 12 min, and ABC reagent (avidin and biotinylated horseradish peroxidase complex) for 30 min (ABC kit; Vector, Burlingame, CA, USA). Detection was accomplished with diaminobenzidine substrate solution until the desired staining intensity was obtained (3 to 5 min). The sections were then counterstained with hematoxylin and eosin, cleared, and mounted.

Quantitative evaluation of Ki-67 expression and TUNEL positivity was performed in epithelial cells from the anatomically normal mammary gland (without any fibrocystic changes), including central and peripheral ducts within the lobules; myoepithelial and lymphoid cells were excluded. Cells were counted as apoptotic only if they were TUNEL-positive and showed characteristic nuclear morphology typical of apoptosis (that is, cells containing pyknotic nuclei plus apoptotic bodies). Successive counts, performed by individuals blinded to the groups, were made until 1,000 cells per tissue sample had been examined. Two indices were thus obtained: the proliferation index (PI), defined as the number of Ki-67-positive nuclei per 1,000 epithelial cells, and the AI, defined as the number of TUNEL-positive cells per 1,000 epithelial cells counted. From these values the cell renewal index (CRI = PI/AI) was obtained.

### Statistical analysis

Statistical analyses were performed with analysis of variance for continuous data followed by Student's *t*-test, and with non-parametric tests (Mann–Whitney *U *test) for non-continuous data (gestation and parity). Fisher's exact test was used to verify the homogeneity of the samples relative to nursing history. The variation in frequency of proliferation and apoptosis relative to variations in the menstrual cycle as well as the variation in frequency of proliferation and apoptosis relative to the progesterone levels were measured with linear regression models and fourth-degree polynomial curves (Polnom Program; University of Manchester, Manchester, UK) as indicated. All data are expressed as means ± SD; *P *< 0.05 was considered significant.

## Results

There were no differences between patients in group A (follicular phase) and group B (luteal phase) in terms of age of menstruation onset (12.8 ± 1.3 and 12.7 ± 1.4, respectively), number of pregnancies, parity, and lactation history.

**Figure 1:**
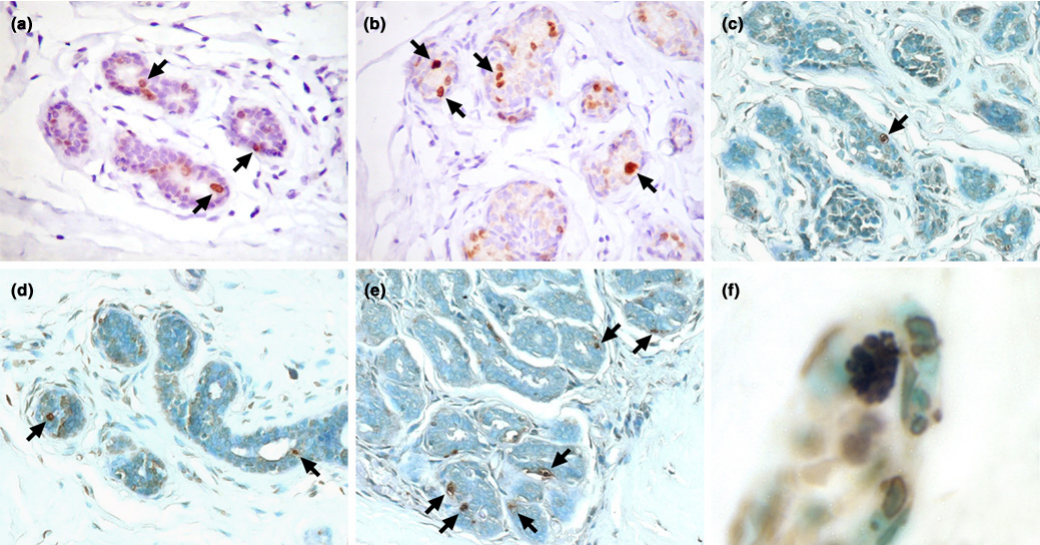
Photomicrographs of human mammary tissue during the follicular and luteal phases of the natural menstrual cycle. **(a,b) **Ki-67 expression (arrows and/or deep brown color) in epithelial cells from normal mammary lobule of a representative patient in the follicular phase (a) and in the luteal phase (b). **(c–f) **TdT-mediated dUTP nick end labelling (TUNEL)-positive staining (arrows, deep brown color) indicating apoptotic epithelial cells from representative patients in the follicular phase (c,d) and in the luteal phase (e,f); (f) high magnification of a labeled cell displaying the characteristic features of chromatin condensation and apoptotic bodies.

The mean PI for group A was 13.5 ± 9.8, significantly smaller than in group B, which had a PI of 30.5 ± 22 (*P *= 0.003, by Student's *t*-test). Photomicrographs from representative patients showing Ki-67 expression for both groups are shown in Fig. 1a,b. The AI for both groups was very similar: group A (4.4 ± 1.8) and group B (5.2 ± 2.4; *P *= 0.21). TUNEL-positive cells from representative patients for each group are given in Fig. 1C–E; Fig. 1f shows a highly magnified TUNEL-positive cell displaying the characteristic features of chromatin condensation and apoptotic bodies. The CRI for group A (3.8 ± 3.4) was also significantly smaller than that for group B (7.4 ± 6.6; *P *= 0.03).

**Figure 2:**
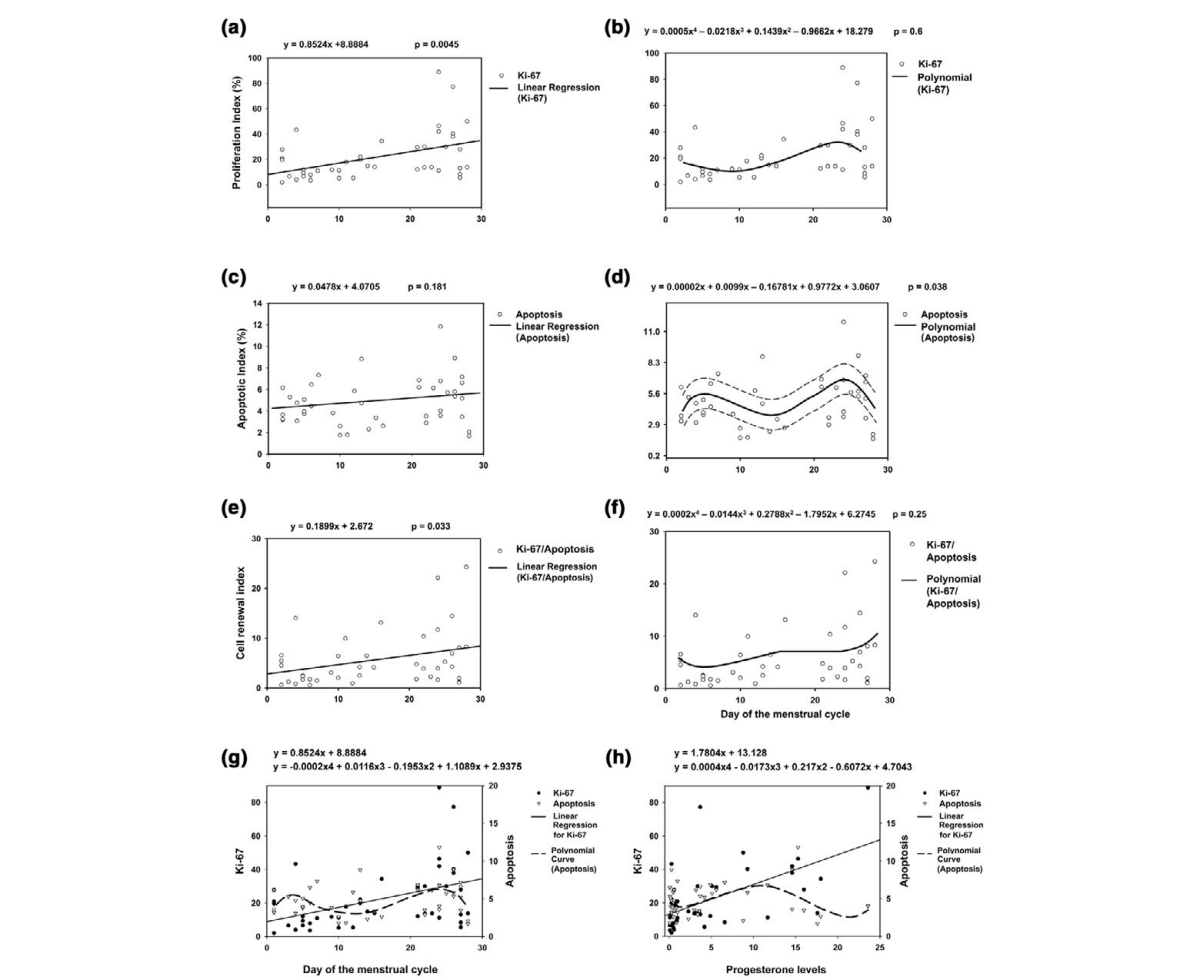
Linear regressions and polynomial curves for the proliferation index **(a,b)**, apoptotic index **(c,d) **and cell renewal index **(e,f)**. The graphs that best represent the proliferation and the apoptotic indexes were the linear regression (a,c,e) and the polynomial curve (b,d,f), respectively. **(g) **By superimposing the data after mathematically adjusting the mean visible time of Ki-67 expression [53] with that of the apoptotic index, one confirms that the maximum values for both indices coincide at about day 24 of the menstrual cycle (the point at which the linear regression and polynomial curves are tangential). **(h) **Correlation of progesterone levels with apoptosis and proliferation irrespective of the stage of the menstrual cycle.

The data show that, in a 28-day interval, the number of proliferative, apoptotic, and cell renewal events vary as a function of time. The linear regressions for each one of the indices are shown in Fig. 2a,c,e. The cyclical variability shows that the highest proliferation values occur near the end of the menstrual cycle, whereas the AI is greatest at the beginning and the end of the menstrual cycle. The CRI shows cyclical variations, with significantly greater values near the 28th day of the menstrual cycle (*P *= 0.033). The fourth-degree polynomial curves show that neither the PI (*P *= 0.6; Fig. 2b) nor the CRI (*P *= 0.25; Fig. 2f) is statistically different throughout the course of the menstrual cycle. In contrast, there is a statistically significant cyclical variability in the AI (*P *= 0.038; Fig. 2d). The mathematical equations that best represent the PI and AI are the linear regression and the fourth-degree polynomial curve, respectively. When these are superimposed, after mathematical adjustment of the median visible time of Ki-67 expression with that of apoptosis, it is clear that the maximum values for both indices (PI and AI) coincide at about the 24th day of the menstrual cycle. At this point, the linear regression for the PI is tangential to the fourth-degree polynomial (Fig. 2g).

Furthermore, progesterone levels, irrespective of the stage of the cycle, correlate with proliferation. In apoptosis this is not so, because we found a decrease in apoptosis at progesterone levels higher than 15 ng/ml (Fig. 2h).

It is important to note that, relative to the total number of cells present, the number of epithelial cells undergoing proliferation or apoptosis at any given time during the menstrual cycle was quite small, and the cells were distributed throughout the mammary lobules.

## Discussion

The mammary gland undergoes cyclical changes in response to the hormonal fluctuations of the menstrual cycle. The epithelium responds to these systemic hormonal changes by regional proliferation, differentiation, and programmed cell death, also known as apoptosis [18]. In the present study of normal mammary tissue we observed that this epithelial response is limited to a small fraction of the cells, suggesting that there is probably local regulation of cell survival and death in this tissue. Similar results were obtained by Andres and Strange [18], who also noted that cells undergoing proliferation or apoptosis were isolated, distributed in various mammary lobules, and positioned close to the lumen.

Proliferation and apoptosis in normal breast tissue are influenced by several factors, including phase of the menstrual cycle, chronological age, breast age, use of oral contraceptives (especially if nulliparous) and recent parity [19]. It is therefore imperative to use strict criteria when selecting patients for studies of normal mammary tissue. Several previous studies have examined cell turnover in morphologically normal human mammary gland epithelium [4-8,16,20-22]. However, interpretation of the results of these studies is difficult for four reasons: first, the menstrual and parity status of the groups were not reported [23]; second, the studies included patients of perimenopausal and/or menopausal age [5,8,23-25]; third, several of the patients studied were using oral contraceptives [5,8,24,26]; and fourth, there are no available data about, or there was no assessment of, the phase of the menstrual cycle with progesterone levels [5,8,23,24,26]. In the present study, all of the above parameters were evaluated and/or controlled for.

As reviewed by Brown and Gatter [27], the PI of any tissue is determined by the growth fraction, which can be assessed by Ki-67 expression, and the time it takes the cell to complete the cell cycle. Thus, tissues in which many cells are in a very long cycle show extensive Ki-67 expression but not a very large PI. In contrast, tissues in which a few cells are in a very short cycle have a higher PI but few Ki-67-positive cells. Because Ki-67 is expressed throughout the cell cycle (G1, S, G2 and M phase), is quickly degraded and is not found in cells undergoing DNA repair [12], its expression provides information only about whether a cell is in the cycle, but not about the cycle length. In accordance with previous studies [5,20], we find that the PI (the number of Ki-67-positive nuclei per 1,000 epithelial cells) of the human mammary epithelium is significantly greater in the luteal phase of the menstrual cycle, both when evaluated by the average of the means and when measured by linear regression.

The mechanisms by which steroid hormones stimulate mammary epithelium growth are controversial and continue to be the source of intense investigation. Various studies suggest that the regulation of cell proliferation occurs via three main mechanisms, namely receptor-mediated [28-36], an autocrine/paracrine loop [37], and negative feedback [38,39]. Among the specific hormone receptors involved, two estrogen receptors (ERs) are of particular interest: ER-α, the predominant subtype in the mammary gland, and ER-β [40]. In normal mammary tissue, the fraction of epithelial cells that express ER is small [41]. Recent studies have shown that the ER is a ligand-dependent transcription factor, which accounts for the latency of most estrogenic responses in target tissues [42]. The ER can regulate gene transcription either directly or indirectly and, depending on the patterns of co-regulator recruitment to the ligand–receptor–gene assembly, can elicit either the stimulation or inhibition of specific biological effects [43]. Additionally, binding of estrogen to the ER can be modulated by progesterone [44]. Acting through the progesterone receptor, progesterone is the physiological negative regulator of estrogen activation [45].

It is possible that steroid hormone fluctuations influence the activation of gene transcription in target mammary cells. These target cells express different types and levels of regulatory proteins in the first and second phases of the menstrual cycle. They require a latency period to initiate the transcription of regulatory factors that activate the cell cycle, culminating with greater proliferation in the second phase of the menstrual cycle.

In the breast, proliferation and apoptosis of the mammary epithelium occur in response to estrogen and other hormones. One of the classical examples of apoptosis is the involution of the post-lactating mammary gland, in which the loss of lactogenic hormones results in collapse of the gland due, in large part, to a programmed elimination of differentiated mammary epithelial cells [46]. In the present study we observe that the apoptotic activity does not vary significantly between the luteal and follicular phases. Moreover, our results show that apoptosis reaches maximum values in the middle of the luteal phase (at about day 24 of the menstrual cycle), but there is also a small elevation in TUNEL-positive nuclei at about the third day of the cycle. This is in contrast to the study by Ferguson and Anderson [5], who found significant cyclical variations in the apoptotic frequency. The reason for the discrepancy in results is unclear but could be related to differences in methodology and patient selection criteria. It is also important to note that in the study by Ferguson and Anderson [5] the significance in cyclical variations is only evident in the logarithm of the transformed, or mathematically adjusted, values for the mitotic and apoptotic frequencies against day of the menstrual cycle. In the present study there is no need to transform any of the values obtained, and we find that the curve that best fits the results is the fourth-degree polynomial.

It is also likely that cyclical variations take place in the rate with which mitotic and apoptotic events occur, thus affecting the time periods at which these phenomena are visible [47]. The outliers in the data may be related to this phenomenon.

The number of events and the rate at which they occur have not been concomitantly evaluated. It is therefore possible that the increase in events during the luteal phase of the menstrual cycle is simply due to the fact that these events are visible for longer during that phase, and not because the absolute number of events is greater at that time relative to the follicular phase. Finally, the increase in events in the luteal phase may be due to a combination of an increased visible time and an increased number of events. Further studies with multi-parameter analysis, allowing us to differentiate between event numbers, duration, and rate of appearance, are needed to understand the cyclical effects of gonadal steroids on breast homeostasis.

Mammary tissue is one of the best examples of the complexity of the biological relationship between cellular proliferation and cell death. The proliferation and apoptotic rates in the mammary gland vary numerous times throughout life depending on age, phase of the menstrual cycle, parity, puerperal involution, and menopause. To study the dual effect of proliferation and apoptosis in mammary epithelial cells, we use the CRI (calculated as PI/AI). We find that, although both proliferation and apoptosis occur throughout the menstrual cycle, the maximal CRI values occur at about the 28th day of the cycle. It is important to note that the isolated comparison of the apoptotic and proliferation indices has some limitations, because each index reflects both the number of times that each event takes place in unit time and the proportion of cells in the population capable of undergoing each event [48]. The CRI allows us to measure the frequency of occurrence of proliferation relative to apoptosis. This serves to reduce or eliminate the effects of differences between samples; cells that do not undergo cellular proliferation or apoptosis do not contribute to the calculation of the CRI [48].

In the present study we find that progesterone levels, irrespective of the stage of the menstrual cycle, are positively correlated with the PI, as seen in animal models [49]. However, the AI decreases with progesterone levels of more than 15 ng/ml, suggesting that mammary epithelial apoptosis is probably dependent on more than just alterations in progesterone levels.

Aside from hormonal fluctuations, there are several factors that change during the menstrual cycle. For example, several Bcl-2 family (apoptosis-regulating) proteins are expressed in the normal mammary epithelium, including the anti-apoptotic proteins Bcl-2, Bcl-X, and MIC-1, and the pro-apoptotic Bax. It has been shown that the relative ratios of these various pro-apoptotic and anti-apoptotic members of the Bcl-2 family determine the ultimate sensitivity or resistance of cells to diverse apoptotic stimuli [46]. Ferrieres and colleagues [50] found a correlation between Bcl-2 expression and progesterone levels, with greater Bcl-2 expression in the follicular phase, diminishing as the menstrual cycle progresses. In contrast, Sabourin and colleagues [51] found that Bcl-2 was expressed preferentially in lobular epithelial cells, with maximal expression at the mid-cycle period and a sharp decrease at the end of the menstrual cycle. Despite the different patterns of Bcl-2 expression in the first part of the menstrual cycle, the results from those two studies suggest that the regulation of Bcl-2 expression in breast tissue is hormone-dependent. Furthermore, as discussed by Ferrieres and colleagues [50], these results suggest a loss in the control of Bcl-2 by progesterone in diseases originating from epithelial lobular components.

Another protein that is also under hormonal control and undergoes cyclic variations throughout the menstrual cycle is epidermal growth factor receptor (EGFR). EGFR is involved in controlling proliferation and, probably, the differentiation of normal breast epithelial cells. Gompel and colleagues [52] have shown that EGFR expression in mammary epithelial cells is stronger in the luteal phase than in the follicular phase. This suggests an effect of progestins similar to that observed in breast cancer cell lines, although it is unclear whether high EGFR levels correlate with higher proliferation or with tissue differentiation.

## Conclusion

Our data indicate that the numbers of proliferative and apoptotic events vary during the menstrual cycle. The frequency with which these events occur is also subject to cyclical variations. The PI and the CRI are significantly higher in the luteal phase. The PI and the AI culminate on about the 24th day of the menstrual cycle, whereas the CRI peaks on the 28th day. Elucidating the cyclical effects of gonadal steroids on breast homeostasis is crucial if we are to understand how alterations in breast structure and function during the menstrual cycle can lead to breast cancer development.

## Abbreviations

AI = apoptotic index; CRI = cell renewal index; EGFR = epidermal growth factor receptor; ER = estrogen receptor; PBS = phosphate-buffered saline; PI = proliferation index; TdT = terminal deoxynucleotidyl transferase; TUNEL = TdT-mediated dUTP nick end labeling.

## Competing interests

The author(s) declare that they have no competing interests.

## Authors' contributions

MAHN, the principal investigator, conceived the study, was responsible for all the patient care and surgical procedures, and wrote the manuscript. CMM performed the TUNEL staining and immunohistochemistry, and aided in drafting of the manuscript. RF performed the pathological study. LGAQ helped with the statistical analyses. GRL and ECB provided valuable input into the manuscript. ACPN supervised the project. All authors read and approved the final manuscript.
